# Reiter’s syndrome following intravesical Bacillus Calmette-Guerin therapy for bladder carcinoma: Case report

**DOI:** 10.1016/j.idcr.2023.e01711

**Published:** 2023-02-19

**Authors:** Hajar Aown Allah Alsulami, Omar Al-Nashiwaty, Mahassen Aly Khalifa, Nazar Ulla Syed, Noha Ahmed, Souad Almuthree

**Affiliations:** a King Abdullah Medical city, Makkah, Saudi Arabia; b Blackpool Victoria Hospital, London, UK

## Abstract

Reiter syndrome is an autoimmune condition that develops in as a reactive response to GI or GU related infectious and rarely related to Intravesical BCG. it is a triad of conjunctivitis, urethritis, and arthritis. Here we report the case of a female patient who developed acute Reiter’s syndrome following intravesical Bacillus Calmette–Guerin (BCG) immunotherapy for superficial bladder cancer. After the sixth dose in the maintenance phase of treatment the patient developed conjunctivitis, aseptic urethritis, and polyarthritis consistent with a diagnosis of Reiter’s syndrome. In this patient non-steroidal anti-inflammatory drugs (NSAIDs), oral steroids and anti-tuberculosis drugs were administered with complete resolution of symptoms.

## Introduction

Intravesical Bacillus Calmette–Guerin (BCG) has been proven as effective immunotherapy treatment for superficial transitional cell carcinoma (TCC) of the bladder, especially for high-grade tumors and carcinoma in situ. It has been widely accepted that BCG therapy can decrease the risk of tumor recurrence, and BCG maintenance therapy contributes to a decreased risk of progression for patients with high-grade non-invasive bladder cancer [1]. Side effects associated with BCG instillations, including fever, myalgia, malaise, dysuria, hematuria, and irritable lower urinary tract symptoms. Reiter’s syndrome is a form of arthritis affecting the conjunctivae of the eyes, the urinary tract, muscles, skin, and joints [2] It is a rare type of reactive arthritis and is known to be a rare severe adverse effect of BCG therapy. However our patient developed Reiter’s syndrome following intravesical instillations of Bacillus Calmette Guerin (BCG) for superficial bladder transitional cell carcinoma (TCC).

## Case report

63 year female Patient known case of hypothyroidism on levothyroxine 25 mcg daily and bladder cancer status post trans urethral resection of bladder tumor (TURBT) one and half year prior to her presentation and status post two induction cycles of BCG- first cycle being 1 year ago and second cycle one month ago. Routine out patient follow up, revealed history of fever, haematuria and dysuria commensurate with the last dose of BCG with knee swelling and Lower limb swelling and other systemic review being unremarkable she also developed mobility issues and soon became wheel chair bound. she give positive history of contact with TB 6 years ago. On Examination Patient was conscious and oriented to time, place and person and stable vitals. Eyes examination showed marked conjunctival suffusion. Joint examination upper limbs was essentially normal with normal range of movements in all the joints. Lower limbs examination right knee tenderness on with limitation of extension and flexion, but no obvious inflammatory signs. Initial laboratory investigations revealed no leukocytosis (white blood cell count, 8.6 * 109/L). Results of renal and liver function tests were within normal limits. Urine dip was positive for leukocyte esterase. The chest x-ray was unremarkable, CT KUB with contrast showed the urinary bladder is partially distended and demonstrates symmetrical circumferential edematous wall thickening with mucosal enhancement consistent with BCG-related cystitis**.**

Patient was commenced symptomatic treatment in the form of Ibuprofen with PPI cover as PRN for 5 days and referral to rheumatology clinic. Apparently seen in the clinic with initial impression of Rheumatoid arthritis and started on prednisolone 30 mg daily reducing regimen over 2 weeks only with a good clinical response. Subsequently, 2 weeks into the follow up period her symptoms recurred with similar Right knee joint pain particularly being worse on Flexion and Extension. She was further subjected to an auto Immune work up in the form of RF, ANA and DsDNa which came back as normal and RA ruled out. She was followed up in ID clinic in the ensuing 2 weeks, and reported very minimal clinical improvement wherein she could bear weight and barely walk a few yards. The poor response to the Analgesics and flare up after steroid tapering raised the likelihood of “Reiters Syndrome” secondary to BCG instillation. and the patient was subsequently commenced on anti-tuberculosis medications in the form of Rifampicin PO 10 mg/Kg and Isoniazid PO 5 mg/kg along with Pyridoxine 20 mg PO. The patient then went on to have a Flexible Cystoscopy under urology which showed normal urethra and bladder with no recurrence and plan to follow her with Cystoscopy in first 2 year on 3 months basis and then every 4 months in 3rd year, and every 6 months thereafter until 5 years. Subsequent Follow-up in ID clinic 3 weeks after starting ATT showed very significant clinical improvement and the patient now was independently mobile after completion of Anti TB of 6 months and plan to follow her every 3 months.

## Discussion

In 1976, Morales et al. published the ground breaking results of the first successful clinical trial of superficial bladder cancer treated with intravesical BCG. Although BCG therapy has been evaluated as a possible treatment for many cancers throughout the years, it has become the most effective therapy, and thus the standard of care, for non-muscle -invasive Bladder cancer (NMIBC) only [3]. It showed benefit in eradication of residual tumour, slows the progression of the disease, reduces the need for cystectomy, and prolonged survival [4,5]. Complications induced by BCG can occur and may vary from self-limited irritative voiding symptoms to severe systemic sepsis. According to the results of the largest and most recent published study by the EORTC Genito-Urinary Cancers Group, of the 1316 patients who started BCG, 69.5 % reported with local (62.8 %) or systemic (30.6 %) complications [6]. Chemical cystitis (35.0 %) and general malaise (15.5 %) were most frequent, and a total of 103 patients (7.8 %) stopped treatment because of complications. From a case series and literature review, 282 cases of BCG infection after intravesical instillation were analysed, and the most common presentations included disseminated (34.4 %), genitourinary (23.4 %), and seromuscular (19.9 %) infections [7]. The study conducted by Lamm et al. showed that disseminated BCGitis was extremely rare. Some studies have reported that, among patients with bladder cancer, there is a relationship between the complications of BCG instillation and a prior diagnosis of tuberculosis, such complications not occurring in patients without a history of tuberculosis and with no evidence of sequelae on chest X-rays [8]. Some studies report Reiter’s syndrome following BCG therapy summarized in Table 1. For primary management of Reiter’s syndrome following BCG therapy, systemic inflammation is usually well controlled by discontinuation of induction. In addition, NSAIDs, steroids, antibiotic agents, anti-tuberculosis agents. Generally, patients with mild symptoms respond easily to NSAIDs but our patient not responding well to it as she had severe arthritis. On the other hand, in cases with severe symptoms or unsatisfactory responses to NSAIDs, steroids should be promptly administered. Delayed introduction of steroids may cause a progressive and prolonged disease course [9]. On the other hand, there is no consensus that the use of anti-tuberculosis drugs to manage Reiter’s syndrome following BCG therapy is recommended. Some reports support the efficacy of anti-tuberculosis drugs because Reiter’s syndrome may be a systemic immune reaction caused by chronic BCG infection in the urinary tract. Our patient had "Reiter's syndrome", after completing intravesical BCG dosage, some previous studies report the cases with no evidence of cancer progression after Reiter’s syndrome following BCG therapy [10], [11].

**Table 1:** Review of published cases reporting Reiter’s syndrome following intravesical Bacillus Calmette-Guerin.

Age- gender	No of doses of BCG	Treatment	Publication
39-year- Male	Fourth dose of BCG	NSAID	Ken glim et al. (2014)
60-year- Male	Sixth dose of BCG	NSAID	Kauther ben et al. (2014)
91-year- Male	Fourth dose of BCG	NSAID + prednisone	Daichi Nonomura et al. (2013)
48-year-Female	Eight dose of BCG	NSAID	Noriko Hatano et al. (2011)
49-year- Female	Sixth dose of BCG	Sulfasalazine +prednisone	Cheung et al. (2017)

## Conclusion

The Intravesical BCG is one of the effective treatment for superficial transitional cell carcinoma (TCC) of the bladder.These adverse effects do not always occur after the initial exposure during induction therapy and may consequently happen during maintenance phase. Here we present one of the rare complications of intravesical BCG in a female patient that developed Reiter syndrome after intravesical BCG immunotherapy in the maintenance phase. Patient was closely followed by rheumatology and infectious diseases teams and her symptoms dramatically resolved with antitubercular treatment. Clinicians should be aware of this rare complication of BCG immunotherapy for early diagnosis and management. However Further research is required to delineate the optimal management options of Reiter’s syndrome.

## Ethical approval

Ethical approval No :22-996, by KAMC IR.

## Consent

We don’t include any picture from the patient in our case report.

## Author agreement

All authors have seen and approved the manuscript.

## Author statement

All authors have participated equal and no conflict of interest (writing manuscript, revision of the manuscript).

## Conflict of interest

The authors declare that they have no competing interests
